# Earliest known ophiuroids from high palaeolatitude, southern Gondwana, recovered from the Pragian to earliest Emsian Baviaanskloof Formation (Table Mountain Group, Cape Supergroup) South Africa

**DOI:** 10.1371/journal.pone.0292636

**Published:** 2023-10-25

**Authors:** Caitlin Reddy, Ben Thuy, Mhairi Reid, Robert Gess

**Affiliations:** 1 Geology Department, Rhodes University, Makhanda/Grahamstown, South Africa; 2 Department of Palaeontology, National Museum of Natural History, Luxembourg City, Luxembourg; 3 Department of Earth Sciences, University of Oxford, Oxford, United Kingdom; 4 Albany Museum Makhanda/Grahamstown, Makhanda/Grahamstown, South Africa; Laboratoire de Biologie du Développement de Villefranche-sur-Mer, FRANCE

## Abstract

For the first time, ophiuroids have been found in South African strata predating the lowermost Bokkeveld Group. These comprise natural moulds and casts from two localities in the ‘upper unit’ of the Baviaanskloof Formation (Table Mountain Group). As a Pragian to earliest Emsian age has been inferred for this member, the new taxa comprise the earliest high-palaeolatitude ophiuroid records from southern Gondwana. Morphological analysis of the specimens revealed the presence of two distinct taxa. One is here described as *Krommaster spinosus* gen. et sp. nov., a new encrinasterid characterised by very large spines on the dorsal side of the disc, the ventral interradial marginal plates and the arm midlines. The second taxon is a poorly preserved specimen of *Hexuraster weitzi*, a cheiropterasterid previously described from the slightly younger Bokkeveld Group.

## Introduction

Ophiuroids, commonly referred to as brittle stars, are members of the phylum Echinodermata, are represented by over two thousand species today and are known from a considerable fossil record. The Paleozoic is dominated by what is called ‘archaic’ type ophiuroids. These ophiuroids have an arm morphology very different from that of ‘modern’ type or crown group ophiuroids [1,2]. ‘Archaic’ and ‘modern’ ophiuroids diverged in the Early Ordovician and until recently, ‘archaic’ ophiuroids were thought to have become extinct by the latest Carboniferous [3]. Recent extension of their range, however, indicates coexistence of ‘modern’ form and ‘archaic’ form ophiuroids, until at least the Triassic [4,5]. The decline of the archaic forms is likely related to the mid-Paleozoic Marine Revolution which involved an increase in the diversity of predatory strategies within shallow water environments in low latitudes [6]. ‘Archaic’ ophiuroids are argued to have been more vulnerable to this diversified predation and thus became restricted to communities affiliated with lower predation levels such as marine environments at high latitudes [3]. Currently, the relationship between ‘archaic’ type and ‘modern’ type ophiuroids is poorly understood, as extraordinarily little phylogenetic research and analysis has been conducted for taxa recovered from Paleozoic strata. For this to be resolved, more data is required, specifically from description and revision Devonian and Carboniferous taxa.

**Fig 1:**
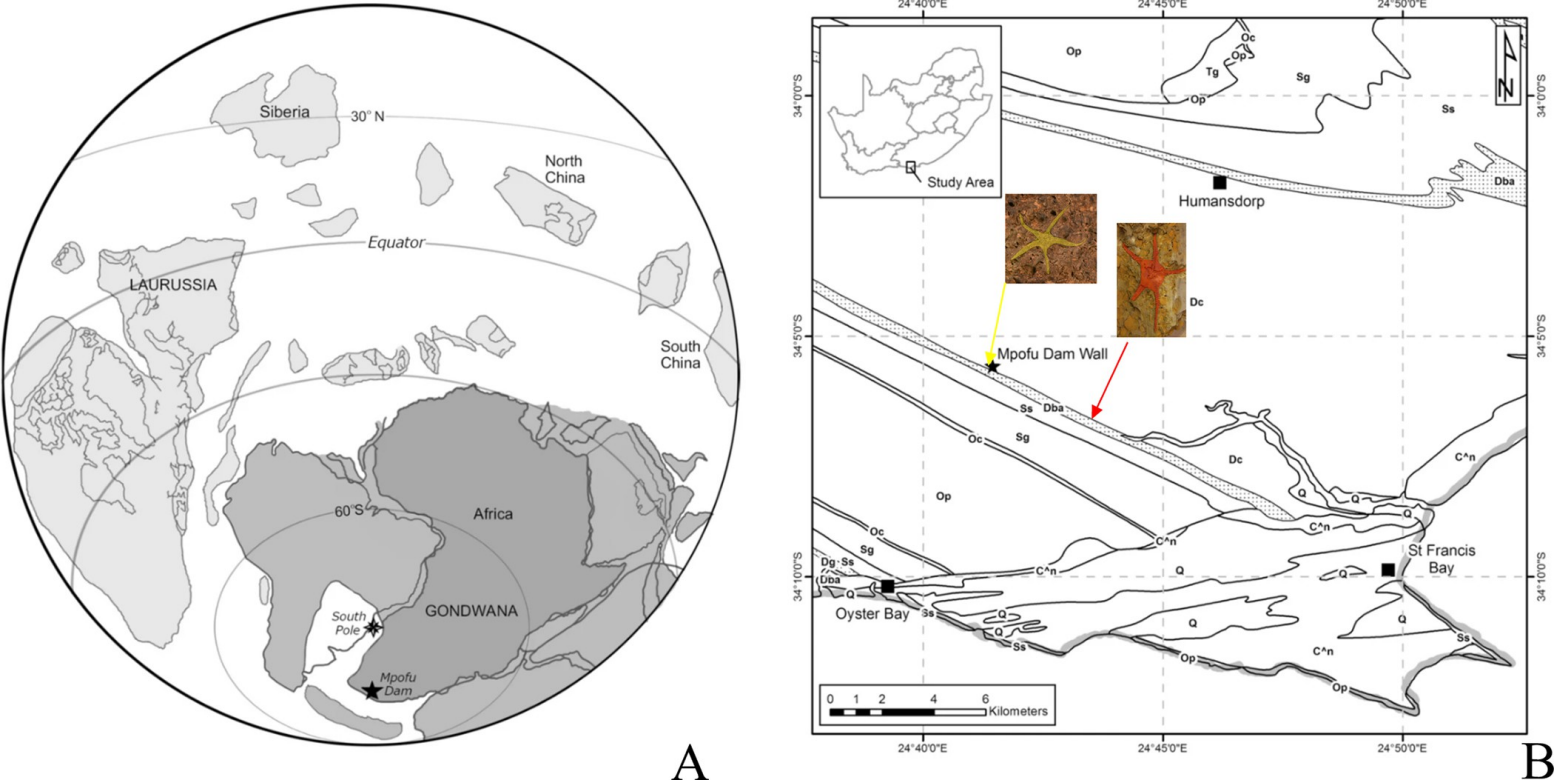
Position of the Impofu Dam and Lag Lens localities. A) Palaeo-geographic location of the Impofu Dam Section locality during the Lower Devonian. B) Current position of Impofu Dam (yellow arrow) and Lag Lens (red arrow) localities. Adapted from Gess and Prestianni [11]. Reprinted from [11] under a CC BY license, with permission from [Robert Gess], original copyright [2021].

Although ophiuroid diversity is comparatively well understood from low palaeolatitude northern hemisphere Paleozoic settings, understanding of biogeographic patterns has hitherto been hampered by paucity of research on Southern Hemisphere deposits. The first extensive account and review of South African Palaeozoic ophiuroids, in addition to other echinoderms such as asteroids, blastoids and crinoids, was provided by Jell and Theron [7], in a revision of reported and unpublished echinoderms from the Bokkeveld Group (Cape Supergroup). Subsequently, Reid *et al*. [8] have described an additional taxon from the Voorstehoek Formation, Bokkeveld Group, which is considered to be Early Devonian in age. Bokkeveld Group ophiuroids comprise part of a well-studied cold water endemic faunal province known as the Malvinokaffric Realm (Malvinoxhosan sensu [9]) [10]. This realm characterises Early to mid-Devonian faunas of southwest Gondwana (Fig 1) also represented by assemblages from southern South America (including Argentina’s Precordillera Formation) and the Fox Bay Formation of the Falkland Islands.

For the first time, ophiuroids have been found in South African strata older than 407.6 mya. These comprise natural moulds and casts collected from two localities in the ‘Upper Unit’ of the Baviaanskloof Formation, the uppermost member of the Table Mountain Group, which underlies the Bokkeveld Group. The taxonomic affinities of these oldest known high latitude (Malvinokaffric) ophiuroids is presented below.

## Geological background

The Table Mountain Group forms the lowermost of three subdivisions within the Cape Supergroup (Braddy and Almond, 1999). Its uppermost three Formations, the Baviaanskloof Formation, Skuweberg Formation and Goudini Formation, are grouped within the Nardouw Subgroup [12]. The Baviaanskloof Formation is the lateral equivalent of the Rietvlei Formation in the west of the depositional basin.

**Fig 2:**
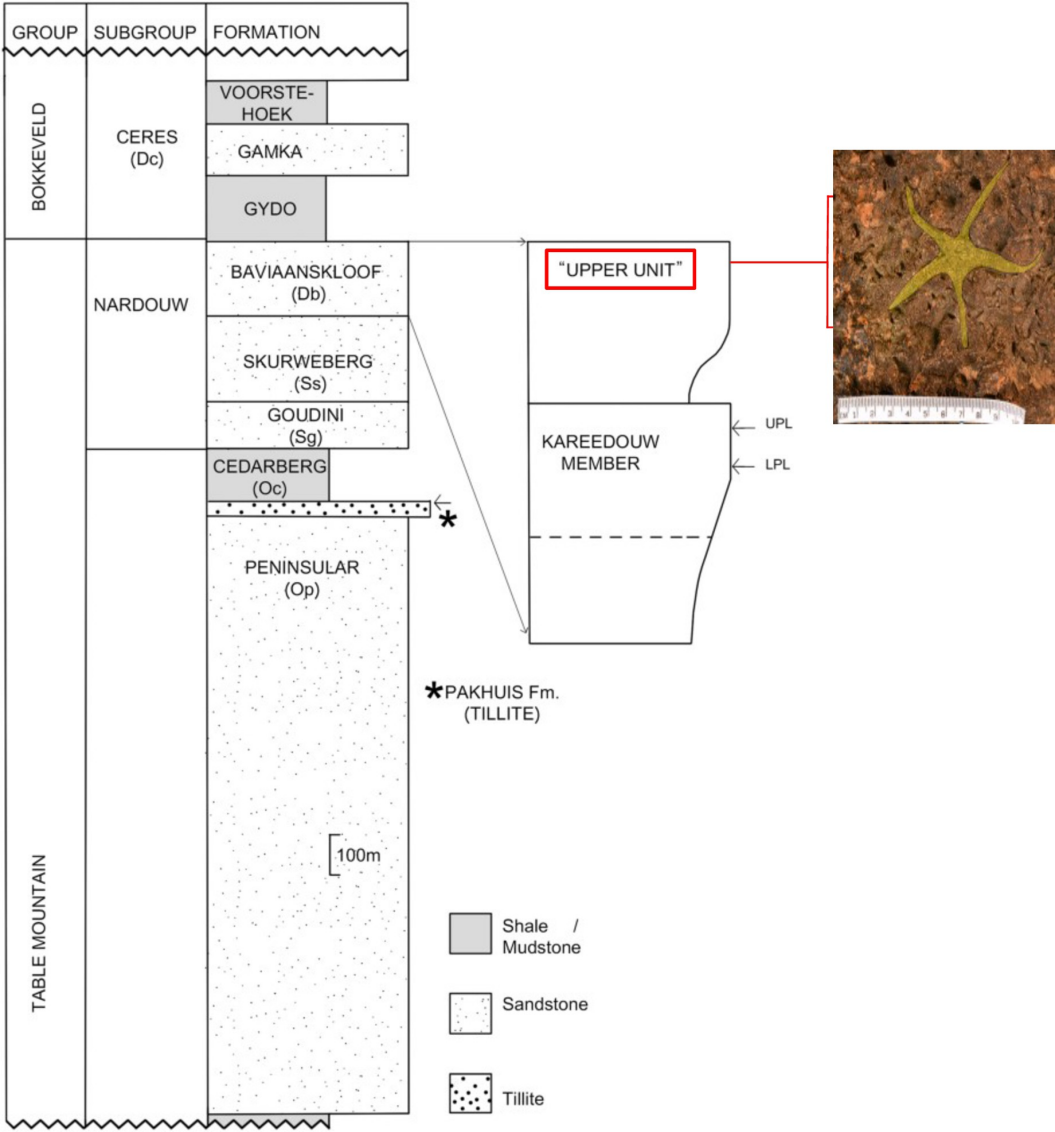
Stratigraphy of the Impofu Dam locality. Modified after Gess and Prestianni, [11], with stratigraphic setting of the Mpofu Dam ophiuroid indicated.

Ophiuroid material herein reported was recovered from two localities within the ‘upper unit’ of the Baviaanskloof Formation (Fig 2).

This unit coincides with initiation of deepening of the Agulhas Sea basin resulting from tectonic subsidence, as well as a rise in global sea level [13]. This engendered a change in depositional setting from a predominately fluvial and shallow shelf environment, during the deposition of most of the Table Mountain Group, to a fully marine depositional environment, during deposition of the overlying Ceres Subgroup of the Bokkeveld Group [11,13]. The Baviaanskloof Formation overlies the Skuweberg Formation, which was fluvially deposited, and is overlain by the fully marine Gydo Formation (Bokkeveld Group [11] (Fig 2). It is predominantly comprised of fine-grained feldspathic sandstones, interbedded with subordinate mudstones [11]. Palaeontological material previously recorded from the ‘upper unit’ includes a range of articulate and inarticulate brachiopods, marine invertebrates comprising homalonotid trilobites, plectonotid “gastropods”, nuculid and other bivalves, as well as bryozoans [11,14–18]. The ‘upper unit’ is underlain by the Kareedouw Member from which diverse, characteristically Lochkovian-aged plant material has been recovered [11]. Marine invertebrates within the ‘upper unit’ are consistent with a Pragian to earliest Emsian age [11], according well with an Emsian attribution of the overlying Gydo Formation [11,19].

A near continuous section through the Baviaanskloof Formation is the subject of ongoing research at Impofu Dam. The stratigraphically lower part of this locality is comprised of thick beds of impure greywacke which then grade upwards into more pure and resistant strata characteristic of the Kareedouw Member. The top of this member is abruptly reached after approximately 60% of the stratigraphic thickness of the formation [11]. Within this lower 60%, the succession of greywacke is interbedded with thin, black, silty, micaceous mudstone lenses, some of which contain plant fossils [11]. Furthermore, it contains no record of marine invertebrates [11].

In contrast, the overlying deposits of the ‘upper unit’ host interbedded, brownish, bioturbated mudstones towards its base, and frequent stringers of brachiopod remains higher in the succession. Mudstones are rich in shallow marine invertebrate remains as well as trace fossils [11]. The ‘upper unit’ is interpreted as representative of shoreface and foreshore sand deposits, with occasional shifts towards preservation of shallow marine muds [11].

**Fig 3:**
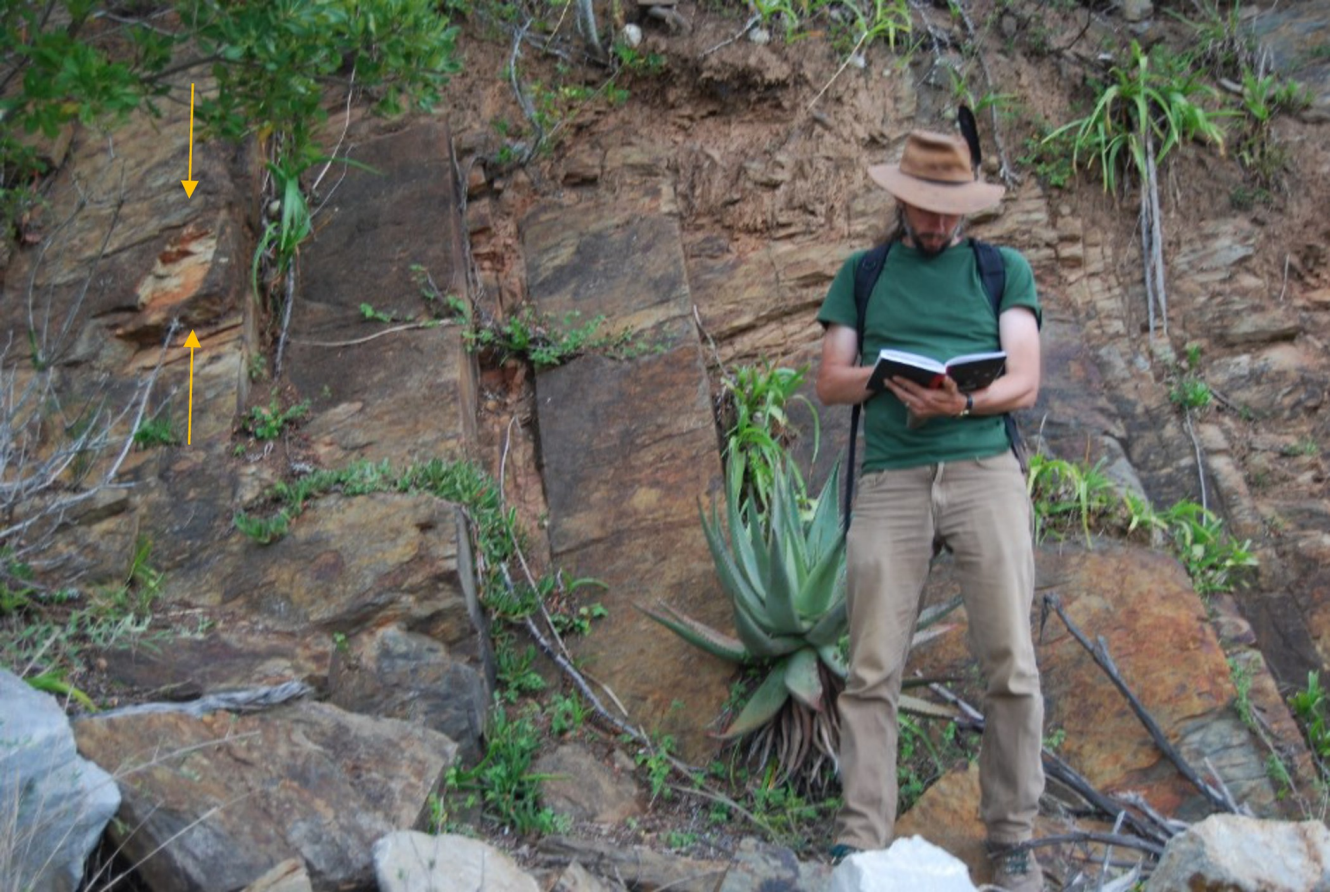
The site from which the Impofu Dam specimen originates. Arrows highlight where in the stratigraphy the specimen was found.

A single specimen comes from the Impofu Dam section (Fig 3). It was recovered from a thin (approximately 1 cm thick) sheet-like monotaxic brachiopod lag deposit. This is one of several coquinite lag deposits occurring at intervals through a sequence of bioturbated muddy deposits and siltstones, probably accumulated at relatively shallow depth. The outcrop is exposed in low cliffs adjacent to the Kromme River. The specimen was preserved intact in life orientation. Lack of a counter specimen precludes determination as to whether it was embedded within the brachiopod deposit or situated on top of it.

**Fig 4:**
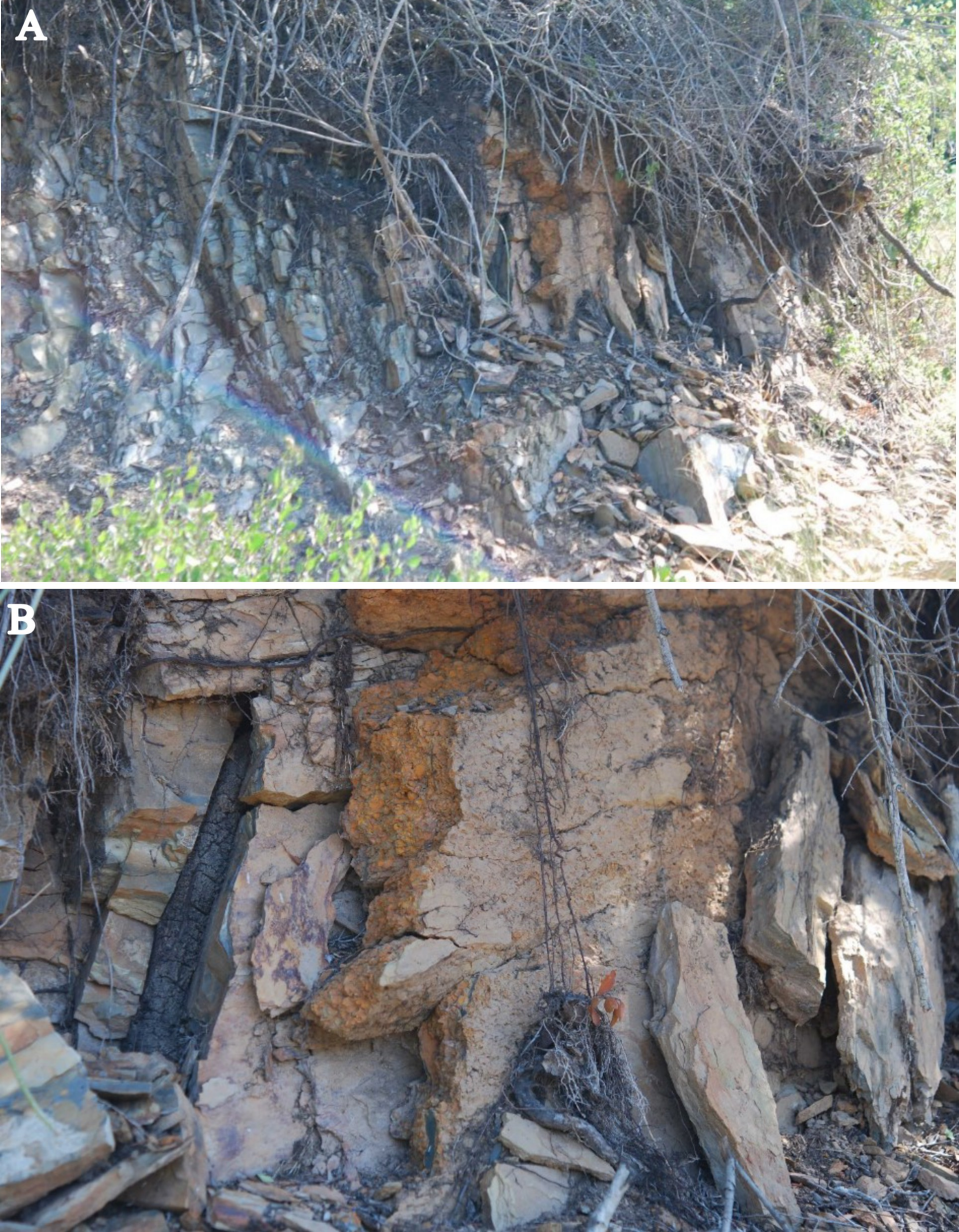
Second locality. A-Lag Lens deposit from the Baviaanskloof Formation. B-The Lag Lens deposit in situ. Stratigraphic top towards the front.

More abundant ophiuroid remains have been recovered from a lens-shaped lag deposit, comprised almost entirely of monospecific articulated brachiopod valves. The latter are complete and articulated, vary in size, and lack preferred orientation. This deposit, encountered in the cut bank of a farm road (Fig 4A and 4B), albeit less well constrained stratigraphically, is clearly situated within the ‘Upper Unit’ of the Baviaanskloof Formation. It appears, however, to be situated marginally higher in the unit than the occurrence at Impofu Dam. The outcrop was exposed in a cutting for a small farm road following the bank of a minor tributary of the Kromme River. The lens, as exposed, was approximately two metres across. It contained rip up clasts of black mudstone, similar to that bedded within the Kareedouw Member, as well as sparse tentaculitids and disarticulated trilobite remains.

Specimens of ophiuroid occur within the mass of shells, usually dorsal side upwards (in normal life orientation), [20], with their delicate arms wound through the shelly matrix. Most specimens were preserved intact. Subsequent to initial deposition the deposit was buried in fine silt which filled the spaces between the organic remains. It has recorded fine internal and external moulds of the original organic components, which have been dissolved during diagenesis. Some ophiuroid specimens were collected in the field but it was considered better to remove large slabs of the deposit for careful systematic disarticulation *ex situ*. The majority of specimens in this study were recovered in this manner. Sixteen specimens have, as yet, been recovered and prepared from the Lag Lens. The majority of the ophiuroids recovered from the Lag Lens deposit are fully articulated, with a few specimens only being represented by disarticulated arms.

## Material and methods

All material was collected by the fourth author, with the permission and under permits granted by SAHRA (the South African Heritage Resource Agency), before being accessioned into the Devonian collection of the Albany Museum, on which the fourth author is the principal researcher.

The lag deposit rocks were first pieced together using a field image and the stratigraphic upper and lower bed surfaces were determined and marked on the rocks. Approximately 30 percent of the lens was systematically disarticulated during this study. Knowing the correct orientation of the rocks permitted establishment of the position of the ophiuroids preserved with the bed, such as dorsal or ventral uppermost. This helps to determine whether the ophiuroids were buried in life position or overthrown before burial.

Once the orientation of the samples was recorded, the rocks were broken using a hammer and chisel and all faunal elements, including ophiuroids, trilobites, bivalves, tentaculites and a selection of brachiopods were placed into designated boxes for later research.

All ophiuroid remains recovered are preserved as empty voids, with all traces of the calcitic skeleton dissolved. In order to study the skeletal details of the specimens, silicone moulds of selected specimens were prepared. To that end, a moat or dam-like structure was formed around the prepared ophiuroid specimens within the rock, using non-curing modelling clay, to prevent the moulding agent from spilling onto surfaces beyond the target area. A releasing agent composed of 2–3 drops of dishwashing liquid and 100 ml of water was mixed in a separate glass and applied to the fossils in order to facilitate removal of the mould whilst limiting damage to the fossiliferous rock. To create the silicone mold, Mold Star 15-Slow™ was used. Equal parts of Mold Star 15-Slow™ Parts A and B were mixed together with a blackened silicone pigment. The silicone mixture was then poured onto the intended specimen from an angle and slight elevation in order to prevent trapping air and the resultant formation of bubbles. The silicone was left to set for 4 to 6 hours. After that, the modelling clay was removed, and the silicone was gently peeled from the specimen. This process was repeated twice as the first peel removed most of the debris and dust from the specimen. The second peel was generally much cleaner and therefore used for analysis.

A gentle and thin coating of ammonium chloride was applied to the peels to highlight details of the specimen. A small quantity of ammonium chloride was placed into the bulb section of a drying tube, filling the bulb to about 2/3 of its volume. The drying tube was then heated over a gas flame until the ammonium chloride was vaporising. The gas was turned then off, and a rubber bulb with a narrow opening (a ‘puffer’) was used to gently blow the vapour out through the smaller end of the drying tube. The silicone peel was held near the exit of the drying tube to receive the ammonium chloride vapour, taking care not to allow the ammonium chloride to ‘cake’ on the peel. The silicone peel was then photographed using a 100m camera lens and low-angled non-polarised light. Photos were then converted into a black-and-white format.

With respect to ossicle terminology and higher-level classification, we align with Gladwell [21]. Unless otherwise stated, ossicle terminology follows that of Spencer and Wright [22], Jell and Theron [7], Glass [23] and Gladwell [21].

### Nomenclatural acts

The electronic edition of this article conforms to the requirements of the amended International Code of Zoological Nomenclature, and hence the new names contained herein are available under that Code from the electronic edition of this article. This published work and the nomenclatural acts it contains have been registered in ZooBank, the online registration system for the ICZN. The ZooBank LSIDs (Life Science Identifiers) can be resolved and the associated information viewed through any standard web browser by appending the LSID to the prefix “http://zoobank.org/”. The LSID for this publication is: urn:lsid:zoobank.org:pub:2C8D3CCE-68E7-4D62-9037-4184EE45F2CF. The electronic edition of this work was published in a journal with an ISSN, and has been archived and is available from the following digital repositories: PubMed Central, LOCKSS.

## Results

### Systematic palaeontology

Class–OPHIUROIDEA Gray, 1840 [24]

Order–OEGOPHIUROIDEA Matsumoto, 1915 [25]

Suborder–LYSOPHIURINA Gregory, 1897 [26]

Family–ENCRINASTERIDAE Schuchert, 1914 [27]

Subfamily–ENCRINASTERINAE Schuchert, 1914 [27]

*Diagnosis—*adapted from [28]: Small to large-sized ophiuroids; ambulacral ossicles alternating, commonly boot-shaped in ventral view; adambulacral plates (= lateral arm plates) subventral, thick and bulky, with broad ventral surfaces, often bearing rows of pustules, and commonly with curved sutures producing rope- like twists, lacking ambulacral groove spines but lateral arm spines present in some taxa; disk large, with concave interradii, commonly bounded by a stout frame of marginal ossicles; podial basins supported by ambulacrals and adambulacrals, tending toward size reduction laterally.

*Krommaster* gen.nov.

*Type species—Krommaster spinosus* sp. nov., by original designation. urn:lsid:zoobank.org:act:ADF93CC7-4213-42C6-B3CF-DF9CFE0244AB

*Diagnosis*—Moderately large encrinasterid with disk covered by a mosaic of small, thin scales and extending to the 5^th^ or 6^th^ arm segment; interradii bound by relatively small marginal plates except for a single larger plate bearing a single very large, conical, pointed spine; similar but slightly smaller spines on dorsal disk and along the dorsal midline of the arms; ambulacrals with a very sharp transverse furrow close to the distal edge of the leg of the boot; adambulacral plates with two to three relatively large, short, conical, pointed lateral arm spines.

*Etmology*—‘Kromm’ From Kromme River, in the canyon of which the ophiuroid lag deposit was recovered. Krom is the Afrikaans word for curve. ‘Aster’, latin meaning star.

*Krommaster spinosus* sp. nov.

Diagnosis and type material same as for genus.

*Etymology*—‘spinosus’, latin for spiny or spiky, referring to the presence of large spines on the central disk and arms.

*Type locality and stratum*—Early Devonian, Pragian to earliest Emsian, ‘upper unit,’ Baviaanskloof Formation, Table Mountain Group, Cape Supergroup, Eastern Cape, South Africa.

**Fig 5:**
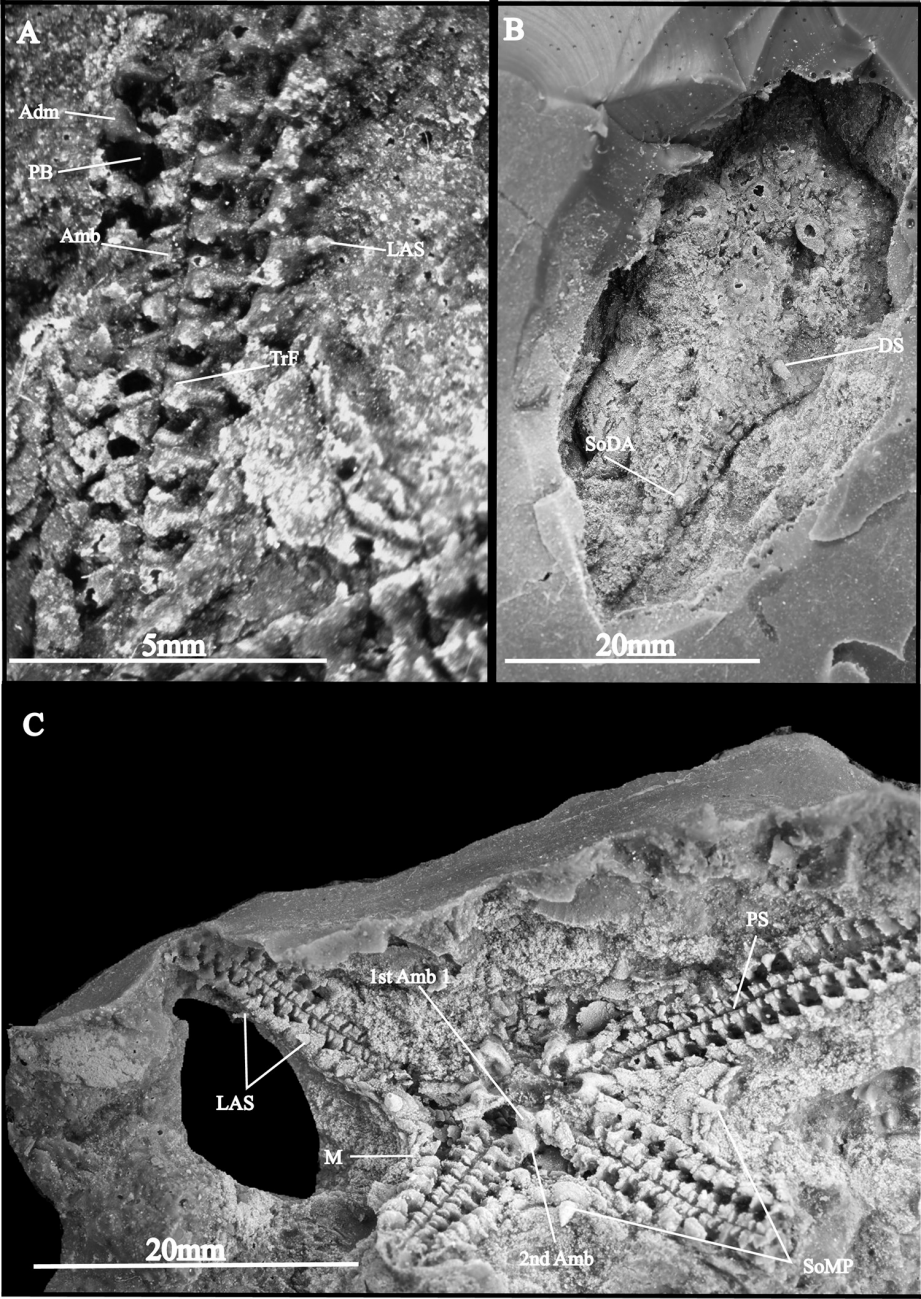
*Krommaster* from the Baviaanskloof Formation, Upper Unit, Cape Supergroup, Table Mountain Group, Eastern Cape South Africa. Holotype AM18222: A) Microscopic close-up of the proximal portion of the arm in the oral view, B) the counter aboral), and C) oral side view of the entire specimen, spines on the disk and arms (Abbreviations: Amb: Ambulacral; Adm: Adambulacral; DS: Disk spine; LAS: Lateral arm spine; MAO: Mouth angled ossicle; PS: Periradial suture; SoMP: Spine on marginal plate; SoDA: Spine on dorsal arm; TrF: Transverse furrow; M: Margin.

**Fig 6:**
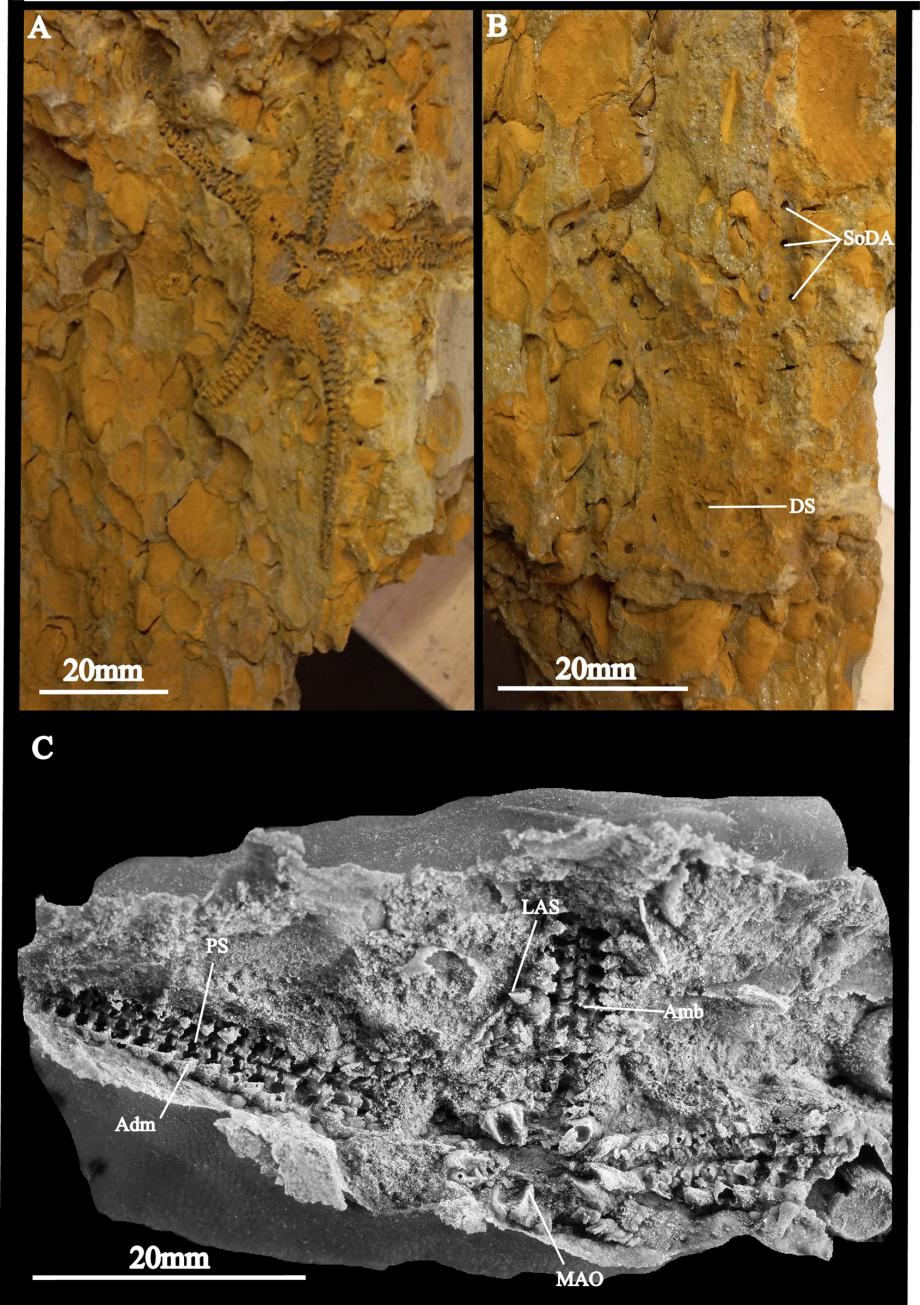
*Krommaster* from the Baviaanskloof Formation, Upper Unit, Cape Supergroup, Table Mountain Group, Eastern Cape South Africa paratypes. A- part aboral view (AM18224) with disk scales preserved, B- counterpart with holes, which comprise moulds of spines (image reversed) (AM18224A), C- silicone peel of the oral view (AM18221). Abbreviations; PS:periradial suture.

*Type material*—Holotype AM18222A+B with ventral and dorsal surface preserved in part and counter-part, (Fig 5B and 5C); paratypes AM18224A+B (Fig 6A and 6B) and AM18221 (Fig 6C).

*Other material* examined—AM18210 –AM18224. All material is housed in the Albany Museum, Makhanda/Grahamstown, South Africa.

*Description*—The holotype (AM18222A, Fig 5) is a fully articulated specimen exposing both dorsal and ventral sides (part and counterpart (AM18222B) of an external mold); disk diameter 20 mm, pentagonal outline with concave interradii; disk covered by a mosaic of small, moderately thick, rounded scales (Fig 5B) a few of which carrying large, short, conical, pointed spines (Fig 5B); interradial margin of disk bound by relatively small, overlapping marginal plates including a much larger central marginal plate with a single very large, conspicuous, conical and pointed spine (Fig 5C) sitting on a circular pit; marginal plates extending to the 5^th^ arm segment; scales on dorsal and ventral interradial areas of disk covered by scattered tiny granules. Oral skeleton only visible in ventral view, formed by large, bulky, short 1^st^ and 2^nd^ ambulacrals (Fig 5C); proximal tips of 1^st^ ambulacrals forming very large, deeply concave recess, one of which showing the ventral tip of a small torus (= dental plate) and possible teeth; 2^nd^ ambulacrals much wider than 1^st^ ones, with distal adradial portions showing a large, deep podial basin and abutting with their adradial tips. Madreporite not observed in specimens.

Arms moderately wide, weakly petaloid, tapering beyond the disk margin; dorsal side of arms covered by small, imbricate scales similar to those on disk, hiding the ambulacrals at least in the proximal arm segments and carrying a longitudinal row of short, conical, pointed spines (Fig 5B) similar to those on dorsal side of disk but smaller; ambulacrals alternating, rectangular in dorsal view; ventral view of arms exposing an obtuse zigzag midline sutural junction between the two rows of ambulacrals; ventral side of ambulacrals (Fig 5A) with a boot-shaped ridge, with a leg as long as or slightly shorter than the width of the foot, a pointed toe with a slightly concave sole, a prominent heel, and a conspicuous ridge bordering the concave distal edge of the leg associated with a sharp furrow (Fig 5A); large, ellipsoid void between the concave distal edge of the leg and the heel of the following ambulacral; podial basins relatively large and deep, enclosed by the lace area of the boot-shaped ridge on the ventral side of the ambualcral and the ventral adradial edge of the adambulacral.

Adambulacrals in ventro-lateral position, not discernible in dorsal view in the holotype specimen; adambulacrals in ventral view (Fig 5A) large, bulky, thick, bulging ventralwards; ventro-adradial edge of adambulacrals with a short, pointed, adradial protrusion articulating with the the toetip of the boot-shaped ridge on the ventral side of the ambualcral; outer surface of adambulacrals with a finely tuberculated outer surface; three large, short, conical, pointed lateral arm spines standing erect close to the distal edge of the adambulacrals, with the ventral spine much larger than the two dorsal ones; no ambulacral groove spines.

Paratype supplements and variation: AM18221 (Fig 6C) is an articulated specimen exposing the ventral side; slightly smaller than holotype (17 mm disk diameter), otherwise similar. One of the 1^st^ ambulacral pairs showing the ventral tip of a torus with two to three small, slender teeth.

AM18224 (Fig 6A and 6B) is a fully articulated specimen exposing the dorsal and ventral sides, similar in size (disk diameter 20 mm) and morphology to holotype; dorsal side better preserved, showing dorsal disk scales with spines and longitudinal row of spines along the dorsal side of the arms (Fig 6B).

**Fig 7:**
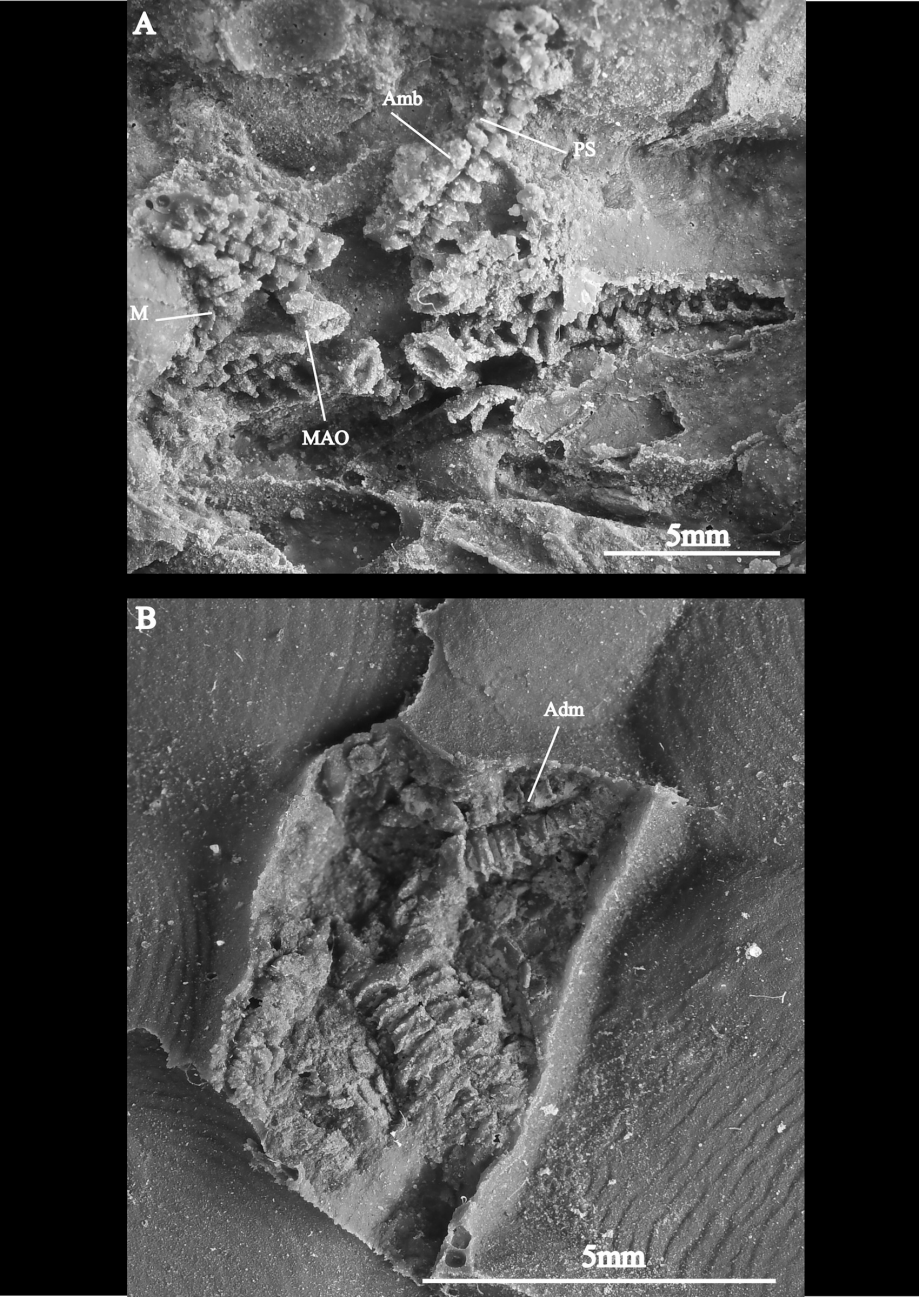
Juvenile specimens of *Krommaster spinosus*. A-(AM18219) oral view of a juvenile B- (AM18220) aboral view.

Small individuals (Fig 7) co-occurring with the specimens above and most probably representing juveniles of the same species are well in agreement with the general morphology of the larger specimens. The main differences are shorter arms, slightly longer ambulacrals, larger 1^st^ ambulacrals with a much a larger proximal recess, and smaller spines on the disk.

*Remarks*—The relatively short, wide, petaloid arms, the large, pentagonal disc with concave interradii bordered by marginal plates, the alternating ambulacrals with a boot-shape ventral ridge, and the large, bulky adambulacrals lacking ambulacral groove spines suggest that the specimens described above belong to the family Encrinasteridae. The presence of relatively large lateral arm spines is unusual in this family as most of the taxa, in particular the type genus *Encrinaster* [29] lack lateral arm spines (e.g. [30]). With *Marginura* [31], however, the family includes taxa that do show well-developed lateral arm spines combined with the typical encrinasterid set of characters.

On the other hand, the specimens described above show characters that are untypical for the Encrinasteridae but instead commonly found in the Protasteridae. The ventral outline of the ambulacrals and of the oral skeleton (1^st^ and 2^nd^ ambulacrals), for example, are strongly reminiscent of some protasterid taxa. Especially the boot-shaped ventral outline of the ambulacrals is typically protasterid in appearance. The conspicuously sharp transverse furrow on the leg of the boot is similar to the transverse furrow found in the ambulacrals of the protasterid *Eugasterella africana* Jell & Theron[7]. In that species, however, the furrow is accompanied by a much more prominent constriction. Spines on the dorsal disk and/or along the disk margin are commonly observed in protasterids including *E*. *africana*. Carinal spines along the dorsal midline of the arms are known from some protasterids, in particular *Strataster* [32], as shown by Hotchkiss [33]. The complete absence of ambulacral groove spines in the specimens described above, however, precludes assignment to the Protasteridae.

With the evidence currently at hand, we favour an assignment to the Encrinasteridae. The unique combination of characters, in particular the conspicuously large, conical, pointed single spine on the central interradial plate, the large, short, conical lateral arm spines, and the sharp transverse furrow on the leg of the ambulacral boot are unknown from any other ophiuroid. We therefore introduce the new taxon *Krommaster spinosus* gen. et sp. nov. to accommodate the material described herein.

Family CHEIROPTERASTERIDAE Spencer, 1934 [34]

*Diagnosis*—Ophiuroids with alternating, cylindrical ambulacrals, very wide adambulacrals devoid of ambulacral groove spines and, in most cases, lateral arm spines, a very large disk lacking dorsal scales and extending to a very close to the tips of the arms.

*Hexuraster* Jell & Theron, 1999 [7]

*Type species*—*Hexuraster weitzi* by original designation

*Diagnosis—*Modified from [7], body large, disk uncalcified, extending at least to the distal third of the arms, sometimes even to their tips. Arms slightly petaloid. Ambulacrals cylindrical with lateral projection (or toe of boot) shorter than radial length. Adambulacrals wide (ad-ab), expanding abradially to be T-shaped, with single very strong lateral spine of each adambulacral. Madreporite interradial, close to mouth frame on ventral side. Mouth frame small for size of animal, plates not massive, with well-developed podial basin on 2^nd^ ambulacral. Mouth large, with short buccal slit between 1^st^ and 2^nd^ ambulacrals.

*Hexuraster weitzi* (Spencer, 1950 [22])

**Fig 8:**
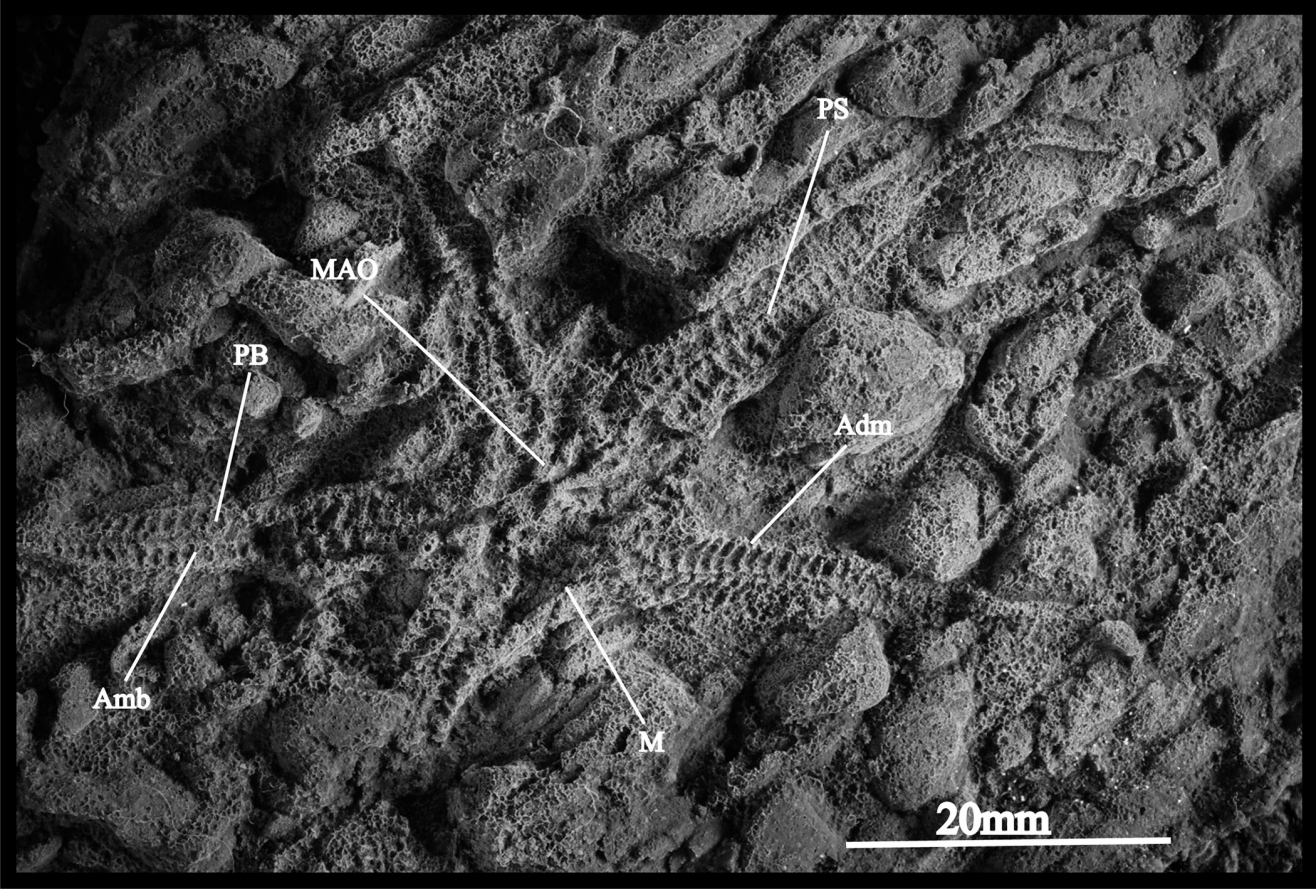
*Hexuraster weitzi* (AM18223) from the Table Mountain Group (Baviaanskloof Formation), Eastern Cape, South Africa. **Ventral view**. A general view of the proximal and distal arm portions with arm details in the oral (ventral) view.

*Material examined*—AM18223 (Fig 8). Material is housed in the Albany Museum, Makhanda/Grahamstown, South Africa.

*Description*—AM18223 is a poorly preserved, fully articulated specimen displaying the ventral side; disk very large, lacking scales, extending at least to the distal third of the arms; arms slightly petaloid, moderately long, ambulacrals boot-shaped in ventral outline, with a cylindrical leg and a long, slender foot; water vessel canal relatively sharp, well defined; adambulacrals very wide, with a long adradial projection articulating with the toe of the boot-shaped ventral side of the ambulacrals and forming very large podial basins; head of the adambulacrals subcircular, lacking ambulacral groove spines, single lateral arm spine discernible in a few arm segments; mouth skeleton poorly preserved, with slender 1^st^ and 2^nd^ ambulacrals. Madreporite not observed in specimen.

*Remarks*—In spite of the poor preservation, the specimen described above shows all the characters of *Hexuraster weitzi*, previously known from the slightly younger Bokkeveld Group. We therefore assign the present specimen to *H*. *weitzi* and thereby expand the stratigraphic range of the species to the Pragian to earliest Emsian.

## Discussion

### Taphonomy of ophiuroids from the Lag Lens deposit

Taphonomic studies show that, prior to decay, complete disarticulation of ophiuroids occurs after seventeen days [35]. Through taphonomic experiments on recently deceased ophiuroids, Kerr and Twitchett [20], derived a matrix for determining the likely time between death and final burial of fossil ophiuroids, based on their degree of disarticulation. Experiments considered both the effects of decay and turbidity currents [20]. Kerr and Twitchett [20] were able to distinguish between ophiuroids that were alive during burial from and those that were deceased before burial occurred [20].

Of the sixteen ophiuroids recovered from the Lag Lens deposit the majority fall within Kerr & Twitchett’s [20] Group 1, i.e. fully articulated specimens with arms preserved and very minor evidence of disarticulation such as the absence of distal arm tips, with over half interpreted to have been fully articulated at time of burial. According to the scheme of Kerr and Twitchett [20] these (Group 1 specimens) are inferred to have been buried alive. Further support for this derives from the preservation of 4 ophiuroids which are in life position. Some of the larger specimens are exceptionally well preserved with distal parts of the arms intact and even the smallest appendices such as disk spines preserved intact (Fig 6A and 6B).

It is therefore probable that these individuals were feeding or sheltering within the brachiopod shell deposit at the time of its burial and were preserved *in situ*. As such, the assemblage can be inferred to represent an autochthonous or at least parautochthonous ophiuroid community.

### Population structure

**Fig 9:**
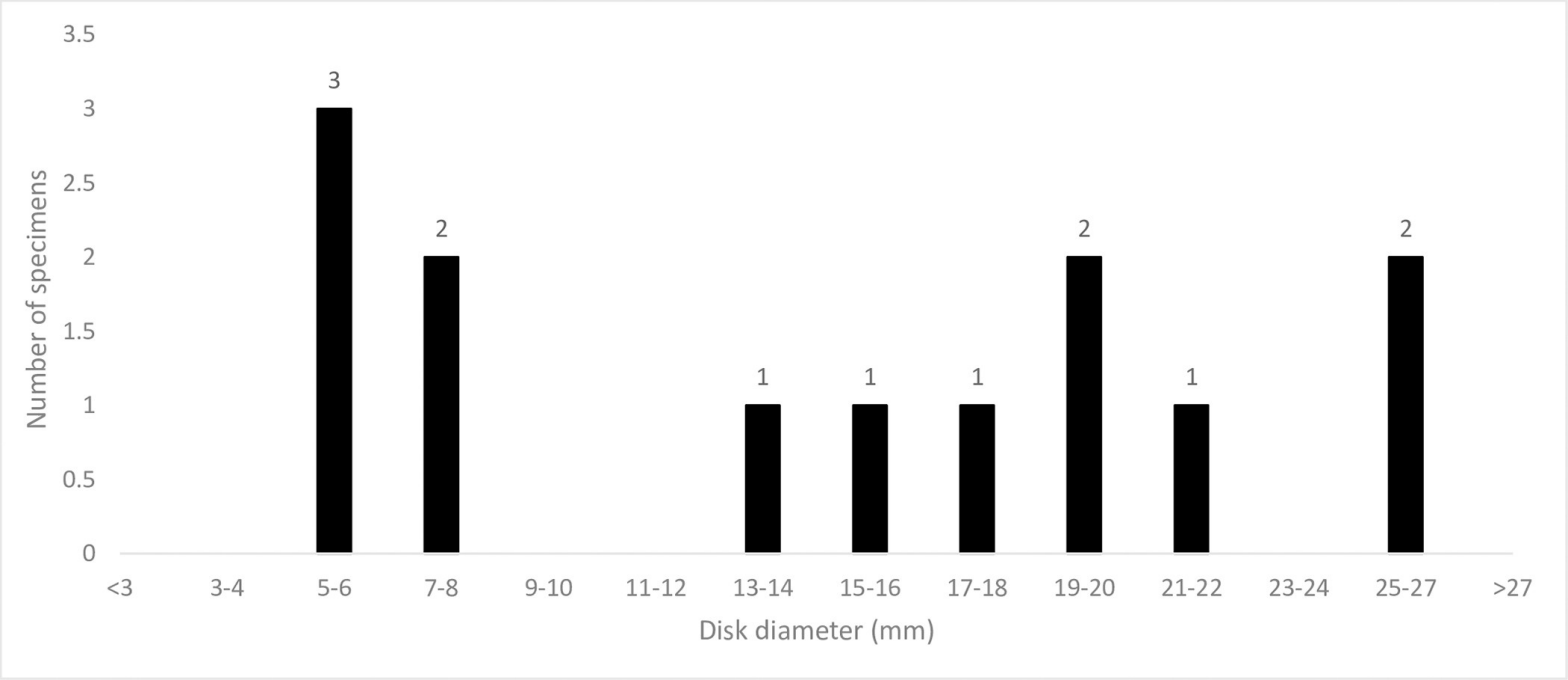
Estimated disk diameter sizes of 12 *Krommaster* specimens from the Lag Lens deposit.

Within the Lag Lens deposit, all ophiuroids are interpreted as a single taxon, ranging in size from 5 mm to 27 mm in disk diameter (Fig 9).

Specimens with their disk diameters or disk radii preserved were used to determine the size range of individuals in the lag deposit. Around half of the sample falls within a size range having disk diameters of 5 to 8 mm, perhaps representing a single juvenile cohort. The majority of the remainder are 13 to 22 mm in diameter, perhaps representing an earlier generation. Two specimens exceeding 25 mm in diameter were also recorded. This suggests a single population comprised of more than one generation cohabiting within the shelly lag deposit. In extant ophiuroid populations, young ophiuroids are similarly found together with adults (e.g., [36]). For example, *Ophiothrix fragilis* (Family Ophiotrichidae), which live under rocks, have juveniles that remain on the dorsal surface of the adults until they reach a disk diameter size of about 4 mm, after which they remain within the same habitat [37].

### Palaeobiodiversity implications

The genera and species of ophiuroid represented in the Baviaanskloof Formation are the first ophiuroids recovered and described from the Table Mountain Group. As such, they represent the earliest known ophiuroids from the southern Gondwanan Malvinokaffric Realm. The discovery of *Krommaster spinosus* gen. et sp. nov. adds a previously unknown taxon to the list of ophiuroids from the Malvinokaffric Realm (e.g., [8,10]). Furthermore, it adds a new genus to the Encrinasteridae and expands the morphological spectrum of the family towards more spinose forms.
