# Correction: Earliest known ophiuroids from high palaeolatitude, southern Gondwana, recovered from the Pragian to earliest Emsian Baviaanskloof Formation (Table Mountain Group, Cape Supergroup) South Africa

**DOI:** 10.1371/journal.pone.0304622

**Published:** 2024-05-24

**Authors:** Caitlin Reddy, Ben Thuy, Mhairi Reid, Robert Gess

The phrase “upper unit of the” is missing from the title. The correct title is: Earliest known ophiuroids from high palaeolatitude, southern Gondwana, recovered from the Pragian to earliest Emsian upper unit of the Baviaanskloof Formation (Table Mountain Group, Cape Supergroup) South Africa. The correct citation is: Reddy C, Thuy B, Reid M, Gess R (2023) Earliest known ophiuroids from high palaeolatitude, southern Gondwana, recovered from the Pragian to earliest Emsian upper unit of the Baviaanskloof Formation (Table Mountain Group, Cape Supergroup) South Africa. PLoS ONE 18(10): e0292636. https://doi.org/10.1371/journal.pone.0292636.
